# Effect of COVID‐19 on healthcare workers' morbidity and mortality compared to the general population in Kohgiluyeh and Boyer‐Ahmad Province, Iran

**DOI:** 10.1002/hsr2.961

**Published:** 2022-12-12

**Authors:** Mohsen Jalil, Zahra Ashkan, Mohammad Gholamnezhad, Somayeh Jamalidoust, Marzieh Jamalidoust

**Affiliations:** ^1^ Emergency Medical Service Yasuj University of Medical Sciences Yasuj Iran; ^2^ Department of Biology, Faculty of Basic Science Shahrekord University Shahrekord Iran; ^3^ Clinical Research Development, Behashti Hospital Yasuj University of Medical Sciences Yasuj Iran; ^4^ Training Center of Department of Education District 2 Shiraz Fars Province Shiraz Iran; ^5^ Department of Virology, Professor Alborzi Clinical Microbiology Research Center, Namazi Hospital Shiraz University of Medical Sciences Shiraz Iran

**Keywords:** COVID‐19, death, healthcare worker, infection rate, Iran

## Abstract

During the Covid‐19 pandemic, the adverse effects of recent coronaviruses on healthcare professionals cannot be ignored. This study compared the admission rates due to Covid‐19 and characteristics of hospitalized healthcare workers with the general population of Kohgiluyeh and Boyer‐Ahmad (K.B) province. 18546 hospitalized patients infected with Covid‐19 in hospitals in four cities of K.B province were enrolled in this study; of them, 236 (1.27%) patients were healthcare workers. Demographic and clinical data of hospitalized cases due to Covid‐19 infection were collected from August 2020 to September 2021. The underlying diseases were also considered in this study. According to our findings, 55.5% of the hospitalized healthcare workers were male, and 44.5% were female; their mean age was 41.41 years. However, in the general population, hospitalization rates were higher for women than for men (51.2% and 48.8%, respectively). Although the SARS‐CoV‐2 infectivity rate was higher in healthcare workers compared to the general population (68.6% vs. 56.1%), the mortality rate was significantly lower in them (1.7% vs. 3.8%). Fever, cough, Acute Respiratory Distress Syndrome, headache, and myalgia were the most prevalent symptoms in both groups. Among the cases examined in this study, inpatient ones aged 30–40 years and the general population aged over 60 seemed to be more likely to be hospitalized for Covid‐19. The hospitalization rate of healthcare workers during the pandemic follows the same pattern as the general population, but since the start of vaccination, this rate has decreased among healthcare workers compared to the general population of KB province.

## 1 INTRODUCTION

In late December 2019, local hospitals in Wuhan discovered the culprit, a novel coronavirus (SARS‐CoV‐2), using a surveillance method for “pneumonia of unknown etiology” that was built in the aftermath of the 2003 Severe Acute Respiratory Syndrome Coronavirus (SARS‐CoV) outbreak with the goal of allowing early detection of novel infections. The World Health Organization (WHO) announced Covid‐19 a “public health emergency of worldwide significance” on January 30, 2020.^1^, ^2^, ^3^, ^4^, ^5^ In general, coronaviruses are genetically categorized into four primary genera, namely Alpha, Beta, Gamma, and Delta. They mostly cause respiratory and gastrointestinal tract diseases. The first two genera primarily infect the mammals, while the third and fourth genera largely infect the birds. Up to now, six types of human coronavirus have been discovered. The Alpha coronavirus genus includes HCoV‐NL63 and HCoV229E, while the Beta genus includes HCoVHKU1, HCoV‐OC43, Middle East Respiratory Syndrome Coronavirus (MERS‐CoV), and SARS‐CoV. Coronaviruses were not well‐known until the 2003 SARS pandemic, which was followed by the 2012 MERS outbreak and, most recently, the Covid‐19 outbreak.^6^, ^7^, ^8^, ^9^ SARS‐CoV‐2 has a single‐stranded RNA genome that is roughly 30 kb in size and is similar to others. Both structural and nonstructural proteins are encoded in the RNA. Spike glycoprotein (S is comprised of two domains, namely S1 and S2), an envelope protein (E), a membrane protein (M), and a nucleocapsid protein (N) are structural proteins which are all located towards the third end of the strand.^10^ SARS‐CoV‐2 mostly affects the respiratory system and, like other respiratory infections, spreads primarily through respiratory droplets thrown via sneezing and coughing. Fever, cough, exhaustion, and shortness of breath are the most common postinfection symptoms. Additionally, some patients lose their ability to taste and smell. However, additional symptoms such as headache, dizziness, and gastrointestinal symptoms (such as nausea, vomiting, and diarrhea) common.^11^, ^12^, ^13^, ^14^, ^15^, ^16^

Healthcare workers (HCWs) are a particularly vulnerable group for infection due to their frequent and close contact with Covid‐19 patients. Thus, it is critical to adhere to stringent hygiene standards to avoid patient‐to‐staff transmission.^17^, ^18^, ^19^, ^20^ Lack of awareness during the early weeks of the outbreak, insufficient Personal Protective Equipment (PPE) supply and training, insufficient rapid diagnostic testing for Covid‐19, long work hours in high‐risk environments, and ongoing community spread and household exposures have all been reported to contribute to the risk of infection amongst HCWs.^21^, ^22^, ^23^ It is critical to identify the clinical characteristics, outcomes, and risk factors associated with SARS‐CoV‐2 infection among HCWs to prevent the virus from spreading in the hospital setting, especially among high‐risk patients and other workers. This information can play a key role in screening strategies and infection control practices, specifically in places where the disease burden is high and resources and protective supplies are limited. Hence, the present study aimed to determine the effect of Covid‐19 on HCWs morbidity and mortality rate compared to the general population. Additionally, symptoms such as headache, dizziness, and gastrointestinal problems (such as nausea, vomiting, and diarrhea) were assessed.

## 2 MATERIALS AND METHODS

### 2.1 Data collection

The current cross‐sectional study was conducted on 18,546 hospitalized patients, 236 of whom were HCWs from 7 different hospitals in Kohgiluyeh and Boyer‐Ahmad province and hospitalized from August 2020 to September 2021. All the samples were sent and tested at the Reference Laboratory of Yasouj University of Medical Sciences.

In this study, a questionnaire was used to collect the data on age; gender; place of employment (medical team, etc.); Covid‐19 patient risk; pregnancy; underlying conditions such as diabetes, cardiovascular disease, chronic lung disease, chronic neuromuscular disease, kidney disease, and liver disease; presence and types of signs and symptoms; and need for hospitalization due to Covid‐19. The Medical Care Monitoring Center (MCMC) also served as a data source. The study design and proposal were approved by the Ethics Committee of Shiraz University of Medical Sciences (IR.SUMS.REC 1399.1319).

### 2.2 Laboratory procedures

Two sterile dacron swabs from the throat and nose of the patients suspected of SARS‐CoV‐2 infection were used to prepare the samples. The clinical samples were not frozen but were thawed repeatedly. Sina Pure kits (SinaClone Co.) were used to extract the viral genomes from 200 µl of each sample, and the extracted samples were then submitted to viral genome quantification using multiplex TaqMan real‐time Polymerase Chain Reaction (PCR) assay kits (Pishtaz Teb Zaman). The final volume of each PCR reaction for each sample was 20 µl. The test simultaneously targeted a specific area of RdRp (FAM‐labeled) and N (HEX‐labeled) of the viral genome as well as the internal control of RNaseP (ROX‐labeled), using the BIORAD real‐time PCR.

### 2.3 Statistical analysis

Descriptive statistics were employed to categorize the research groups based on SARS‐COV‐2 PCR results. The categorized variables are reported using numbers and percentages. The *χ* ^2^ test was employed to determine statistically significant differences between categorical variables, and *p* < 0.05 was considered statistically significant. All data analyses were carried out using the SPSS software, version 26.

## 3 RESULTS

**Figure 1:**
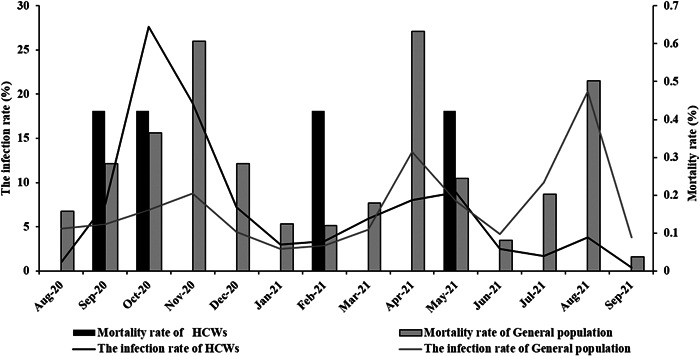
The hospitalization rate of healthcare workers compared to the general population from August 2020 to September 2021, Kohgiluyeh and Boyer‐Ahmad Province, Iran.

This study was done on 18546 patients, of whom 236 (1.27%) were HCWs. Among the HCWs, 20 (8.5%) were physicians (residents and assistants), 116 (49.2%) were nurses, and 100 (42.4%) had other positions in the healthcare system. The hospitalized HCWs worked in seven hospitals of four main cities of this province. During the research period, there were three peaks in hospitalization rate amongst the patients. The highest incidence of SARS‐CoV‐2 infection in the HCWs group was at the first peak with a frequency of 27.6%. Due to more direct exposure to infection, the peak of infection with Covid‐19 occurred earlier in the HCWs than in general population. In the following months, the HCWs' hospitalization rate decreased until the 10th and 13th month when we witnessed an increase again, resulting in the formation of the second and third peaks of hospitalization. (Figure 1). The peak pattern of hospitalization rates for the general population was different from those of the healthcare workers, with the last peak being the highest.

The HCWs' age ranged from 19 to 75 years (mean = 41.41 ± 13.01 years), while the age range of general population was from 19 to 110 years (mean = 51.30 ± 16.67 years).

The hospitalization rate among the HCWs was higher for males than for females (55.5% vs. 44.5%, *p* = 0.093). However, in the general population, the proportion of females was statistically high (51.2% vs. 48.8%, *p* = 0.0012).

A number of participants had known underlying diseases including diabetes, hypertension, cardiovascular diseases, kidney disease, chronic obstructive pulmonary disease, liver disease, chronic blood disease, chronic neurological disease, and immunosuppressive disease. Furthermore, 58.1% of the HCWs and 52.2% of the general population reported that their cause of infection was close contact with confirmed Covid‐19 patients (Table 1).

**Table 1:** Demographic features of the COVID‐19‐infected patients among the general population and HCWs in Kohgiluyeh and Boyer‐Ahmad Province, Iran

Characteristics	HCWs	General population
Age range (years)	19–75	19–110
Sex (%)	—	—
Male	55.5	48.8
Female	44.5	51.2
Pregnancy (%)	2.1	1
Diabetes (%)	7.2	11.2
Cardiovascular disease (%)	4.2	9
Kidney disease (%)	1.7	2
Liver disease (%)	0.4	0.2
Chronic obstructive pulmonary disease (%)	1.3	1.7
Chronic neurological disease (%)	0.4	0.6
Immunosuppressive disease (%)	0	0.2
Chronic blood disease (%)	0.4	0.2
Blood pressure (%)	5.9	16.8
Close contact with confirmed COVID‐19 patient (%)	58.1	52.2

Abbreviation: HCWs, healthcare workers.

**Figure 2:**
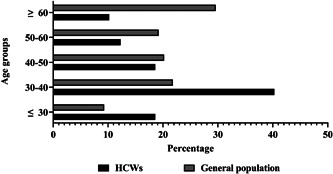
The hospitalization rate of healthcare workers compared to general population in different age groups in Kohgiluyeh and Boyer‐Ahmad Province, Iran.

The most common age group in this study was 30–40 years for HCWs (40.3%) and >60 for general population (29.6%) (Figure 2).

The most common symptoms reported by the hospitalized HCWs and general population were cough, myalgia, Acute Respiratory Distress Syndrome (ARDS), fever, and headache (Table 2).

**Table 2:** Symptomology of the hospitalized HCWs and general population in Kohgiluyeh and Boyer‐Ahmad Province, Iran

Symptoms	HCWs (%)	General population (%)	*p* Value
Fever	35.6	26	**0.001**
Cough	62.7	58.7	0.210
ARDS	48.7	50.7	0.545
Anosmia	4.2	3	0.289
Loss of taste	0.8	1.6	0.372
Nausea	7.6	6.8	0.626
Vomit	5.9	3.7	0.073
Diarrhea	6.4	2.5	**0.000**
Abdominal pain	0.8	2.2	0.157
Anorexia	13.6	14.4	0.702
Chest pain	3.4	4.2	0.545
Headache	22.5	25.4	0.297
Dizziness	3.8	5.1	0.359
Loss of consciousness	1.3	1.7	0.637
Myalgia	54.2	47.9	0.054
Skin lesion	0	0.1	0.562

*Note*: Bold values indicate statistical significance.

Abbreviations: ARDS, Acute Respiratory Distress Syndrome; HCWs, healthcare workers.

As shown in Figure 1, the overall morbidity rate among HCWs and the general population was 68.6% and 56.1%, respectively, and the mortality rate was 1.7% (4/236) and 3.8% (697/18310), respectively. It should be noted that the greater number of deaths in both groups is related to the first, second, and finally third pandemic peaks, respectively (Table 3).

**Table 3:** Treatment modulation for the HCWs and general population and mortality statistics in Kohgiluyeh and Boyer‐Ahmad Province, Iran

Characteristics	HCWs (%)	General population (%)
Oxygen therapy	54.7	42.8
Mechanical treatment	2.1	3
ICU	3.4	4.6
Death	1.7	3.8

Abbreviations: HCWs, healthcare workers; ICU, intensive care units.

The length of hospital stay was 5.77 ± 5.06 days among HCWs and 5.6 ± 5.04 days for the general population. Additionally, 2.1% of the HCWs and 3% of the general population received mechanical treatment. It is worth mentioning that 3.4% of the HCWs and 4.6% of the general population were admitted to intensive care units (ICUs).

## 4 DISCUSSION

HCWs are the most essential human resources in hospitals in the fight against Covid‐19. Infection and death of HCWs endanger not only their lives, but also their morale and can even trigger public panic. HCWs, especially those who work on the frontlines, are frequently exposed to Covid‐19 patients and are, consequently, more susceptible to infection compared to others.^24^, ^25^ The present study showed that the prevalence of Covid‐19 infection in the first 13 months of the outbreak among HCWs and the general population admitted to various hospitals in the Iranian province of KB was 68.6% and 56.1%. The mortality rate in these two groups was estimated at 1.7% and 3.8%, respectively.

Various studies have examined the prevalence of SARS‐CoV‐2 infection among HCWs, which according to PCR testing has been reported at 11% globally; for example, the United States (55%), Mexico (30.35%), and Italy (49.3%) had the highest SARS‐CoV‐2 infection rates. In contrast, Kuwait (20.5%), Egypt (20%), Canada (7%), and Austria reported the lowest SARS‐CoV‐2 infection rates among HCWs.^24^ Another study conducted in Shiraz‐Iran reported that the prevalence of Covid‐19 among healthcare workers was 30%.^25^ The mortality rate in our study, as in Lin and colleagues study, was lower among HCWs than in the general population.^26^

To prevent or at least reduce infection and disease transmission among the HCWs, all necessary measures were taken at the seven hospitals under investigation. The universal use of masks, gowns, gloves, and eye goggles or face shields by all HCWs was regularly practiced in these hospitals. The study findings revealed the incidence of Covid‐19 among 236 HCWs (1.27%), indicating that these individuals were infected as the result of their job. Among HCWs, nurses had the highest infection rate (49.2%); this is in the same line with the results of the research conducted on HCWs in Wuhan, China, reporting a rate of 52% among nurses. This could be due to the fact that nurses averagely spent more time in contact with patients in comparison to other HCWs, such as physicians.^27^ In the same line, Barrett and colleagues revealed a higher prevalence of infection among the HCWs who had direct contact with Covid‐19 patients, with nurses comprising the majority of the affected HCWs (62.5%).^28^ In another study conducted by Lahner and colleagues in Italy, 2057 HCWs were tested by PCR from of March 18 to April 27, 2020 and positive test results were obtained for 58 of them (3%). Among these individuals, 53 (91.4%) had direct contacts with Covid‐19 patients (physicians and nurses), and 5 of them (8.6%) were HCWs with indirect contacts with these patients.^29^ Another research investigated the seroprevalence and relative chances of SARS‐CoV‐2 infection among HCWs employed in various professional categories. In this cross‐sectional investigation on 82,961 serological tests, 12.2% of the Italian HCWs tested positive for IgG antibodies against SARS‐CoV‐2. Additionally, nurses, auxiliary health workers, and the personnel who worked in emergency rooms or were involved in the treatment of patients with subacute diseases had a higher risk of infection.^30^ In the early phase of the Covid‐19 outbreak, the number of HCWs and PPE was insufficient, and the continuous working hours of HCWs were relatively long. As a result, HCWs were physically and mentally exhausted. In this condition, HCWs experienced lowered immunity and had an increased risk of infection. As a result, HCWs on the frontlines are recommended to get enough rest and sleep, avoid overworking, and follow a balanced diet with supplements to maintain proper nutrition. This will boost their immunity and lower the risk of infection in them.^27^

In the current research, males had a higher incidence of infection and hospitalization (55.3%) compared to females, but the difference was not statistically significant (*p* = 0.106). Consistently, Iversen and colleagues conducted a study in Denmark and indicated that the male personnel had a higher prevalence of the condition in comparison to female ones. This was attributed to the unknown underlying patterns of transmission or different behaviors. For example, women might be more cautious in following the healthcare advice.^31^ The data obtained from the Global Health 5050 also showed that the number of Covid‐19 confirmed cases and the death rate of the disease were both high among males in various nations.^32^, ^33^ This might have happened because the behavioral variables and roles that enhance the likelihood of Covid‐19 infection are more common in males. Men are more likely to engage in dangerous behaviors such as drinking, participating in performing the funeral rites, working in basic sectors and occupations requiring them to stay active, working outside their houses, and socializing with others.^34^, ^35^, ^36^

In the current research, the most common symptoms reported by the hospitalized HCWs and general population were cough, myalgia, ARDS, fever, and headache. Similarly, Yang and colleagues mentioned fever, cough, and shortness of breath as the most common symptoms amongst HCWs and general population.^37^

Among the patients under the present investigation, 3.4% of the HCWs and 4.6% of the general population were admitted to ICUs. Evidently, the HCWs were less likely to require admission to ICUs. Moreover, 2.1% of the HCWs and 3% of the general population needed mechanical treatment. Unfortunately, 1.7% of the HCWs and 3.8% of the general population died due to Covid‐19. These results are in the same line with those of the studies carried out by Yang and colleagues and Alshamrani and colleagues which demonstrated a lower need for ICU admission, use of mechanical treatment, and mortality in HCWs than in general population.^37^, ^38^

On February 9, 2021, Iranian Ministry of Health started offering SARS‐CoV‐2 vaccines to HCWs. As mentioned before, there were three maxima in SARS‐CoV‐2 hospitalization during the research period. In the first peak, 27.6% of the HCWs became hospitalized, while the hospitalization rate of general population was 8.8% at that time. In the second and third peaks, however, the hospitalization rates were respectively 8.9% and 3.8% among the HCWs and 13.5% and 20.3% among the general population. The reduction observed in the infection rate of the HCWs could be related to their vaccination as well as their better compliance with health protocols. In the same vein, Bouton and colleagues found that SARS‐CoV‐2 vaccinations reduced the infection rates when the prevalence of the disease was high in the country. According to their findings, the vaccinated HCWs showed a 27% reduction in new cases about 1–14 days after the first dose and an 82% reduction after 15 days compared to unvaccinated HCWs.^39^, ^40^ A reduction in positive test results 14 days after the second dose was also observed in another study, which assessed SARS‐CoV‐2 infection after vaccination in Californian HCWs.^41^ Decreased positive test results postvaccination have also been reported among HCWs in England and other parts of the United States.^42^, ^43^

In conclusion, we found that although the SARS‐CoV‐2 infection rate among hospitalized HCWs was higher than the general population (68.1% vs. 56.1%), the Covid‐19 mortality rate was lower (1.7% vs. 3.8%). SARS‐CoV‐2 infection in the HCWs has shown a decreasing trend over time since the beginning of the pandemic, unlike the general population.

## AUTHOR CONTRIBUTIONS

**Mohsen Jalil**: Conceptualization; data curation; methodology; writing – original draft; writing – review and editing. **Zahra Ashkan**: Data curation; formal analysis; methodology; writing – original draft; writing – review and editing. **Mohammad Gholamnezhad**: Conceptualization; supervision; writing – review and editing. **Somayeh Jamalidoust**: Formal analysis; methodology; writing – review and editing. **Marzieh Jamalidoust**: Conceptualization; data curation; funding acquisition; resources; supervision; writing – original draft; writing – review and editing.

## CONFLICT OF INTEREST

The authors declare no conflict of interest.

## TRANSPARENCY STATEMENT

The lead author Marzieh Jamalidoust affirms that this manuscript is an honest, accurate, and transparent account of the study being reported; that no important aspects of the study have been omitted; and that any discrepancies from the study as planned (and, if relevant, registered) have been explained.

## Data Availability

The data that support the findings of this study are available from the corresponding author upon reasonable request.
